# Infantile Amoebiasis: A Case Report

**DOI:** 10.1155/2012/614398

**Published:** 2012-06-21

**Authors:** Mohammad Zibaei, Farzaneh Firoozeh, Alireza Azargoon

**Affiliations:** ^1^Department of Parasitology and Mycology, School of Medicine, Lorestan University of Medical Sciences, P.O. Box 6814993165, Khorramabad, Iran; ^2^Department of Microbiology and Microbiology Research Center, Pasteur Institute, Tehran, Iran; ^3^Department of Internal Medicine, Shohaday-e-Ashayer Hospital, Lorestan University of Medical Sciences, Khorramabad, Iran

## Abstract

Amoebiasis continues to be a major cause of morbidity and mortality in children in developing countries. *Entamoeba histolytica* infections are commonly observed in tropical and subtropical regions of the world including Iran. In developed countries *Entamoeba histolytica* infections are commonly seen in travelers, recent immigrants, homosexual men, and inmates of institutions. The disease is more severe in the two extremes of life. This paper paper describes a four-month-old male infant with *Entamoeba histolytica* presenting initially with refusal of feeds, hyperactive bowel sound, vomiting, and diarrhea. A fecal sample was positive for *Entamoeba histolytica* by Lugol's iodine solution and the concentration technique. He was successfully treated with metronidazole for 5 days. This case illustrates that *Entamoeba* species could be pathogenic in young infant; therefore, awareness of the infection, aggressive approach to diagnosis, and early initiation of treatment continue to be critical component of infection control.

## 1. Introduction

The causative agent of intestinal amoebiasis is *Entamoeba histolytica* (*E. histolytica*). This parasite is endemic in most tropical and subtropical areas of the world, where it causes millions of cases of dysentery [1]. In a national survey of the prevalence of intestinal parasitic infection in Iran, *E. histolytica* was one of the most common pathogenic protozoa [2]. In endemic areas, breast-feeding and different socioeconomic status do not protect infants from infection with *E. histolytica* [3]. The greatest numbers of infected individuals have parasites restricted to the lumen of the intestine [4]. The persons presenting with colitis typically have a history of several weeks of gradually increasing abdominal cramp, tenderness, and weight loss. A range of bowel function alterations are observed, ranging from frequent mucoid stools to watery and bloody diarrhea, often with periods of dysentery alternating with constipation. An estimated 40,000–110,000 persons die each year from infection with *E. histolytica*, however, and many more are either asymptomatically infected or present with varying degrees of dysentery and extraintestinal diseases [5]. Here, we describe a case of infantile amoebiasis, which, on stool examination, reveals *E. histolytica* trophozoite and cyst as well as intestinal function abnormalities.

## 2. Case Report 

A four-month-old male infant from a rural district in Khorramabad, southwest Iran was brought to the emergency room of the Lorestan University of Medical Sciences Hospital with history of refusal of feeds, hyperactive bowel sound, vomiting, and change in stool texture. The patient also had episodes of retching and mild abdominal distension. The mother suffered abdominal pain and had history of diarrhea.

**Figure 1:**
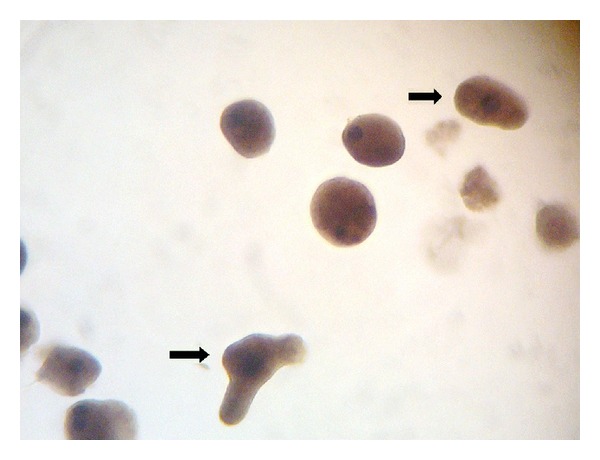
Cysts and trophozoites of *Entamoeba histolytica* (arrows); iron hematoxylin stain of fecal sample, magnification × 1000.

The complete blood count showed a leukocyte count of 7800 *μ*L with eosinophilia of 7%. The results of biochemistry tests were as follows: Na (130.0 mEq/L), Ca (10.4 mg/dL), and total protein (4.6 g/dL) (Table 1). The urine analysis and culture were normal and negative, respectively. Direct examination of fresh fecal samples using Lugol's iodine solution and the concentration technique showed *E. histolytica* cysts and trophozoites with red blood cells (occult blood) and many leukocytes (Figure 1). The infant was given metronidazole syrups (35–50 mg/kg/BW/day) and oral rehydration salt (ORS), which he took for 5 days with improvement. Subsequently, the infant was feeding normally with breast milk. On the seventh day after treatment, the results of the laboratory tests such as CBC and chemistry all were negative. Repeat wet mount prepared on slides from fresh stool samples and stained using iron hematoxylin technique, showed no parasites after examination of many fields of the slides. He was discharged in stable condition 7 days after admission.

## 3. Discussion

Amoebiasis is defined by the World Health Organization (WHO) as infection with *Entamoeba histolytica*, regardless of symptomatology [5, 6]. This protozoan parasite is the pathogenic species responsible for amebic colitis throughout the world. It infects people of both sexes and all ages; however, populations at risk may vary with geographic location, host susceptibility, and differences in organism virulence [7, 8]. Most infections (≥90%) remain asymptomatic, although invasive intestinal disease may occur in days to years after initial infection and is characterized classically by abdominal pain and bloody diarrhea. Watery or mucus-containing diarrhea, constipation, and tenesmus may also occur [9–11].

Spread of the infection occurs due to consumption of food and water that is contaminated with the cyst. In this case, the infection is not endemic to the city of Khorramabad, and the city's water supply facilities are known to be safe. Due to the local custom, family members are having meals together on a common dining plate, which could result in transmission of *E. histolytica *among close contact. The mother had history of amoebic infection and stool exams showed cysts of *E. histolytica*. Therefore, inadequate hand washing by the mother could have resulted in contamination of the expressed breast milk fed to the infant through gavage [12, 13].

Ilikkan and colleagues reported 11 children with acute amoebiasis. Eight of infants were breast fed, and none of them developed extraintestinal disease [3]. Kahng and Kim observed a one-day-old female with vomiting and bloody stool. Her abdomen was soft flat with decreased bowel sound. The patient's vital signs were all normal [14]. A case of amoebiasis in a newborn with vomiting, refusal of feeds, abdominal distension, and mucoid stool, was reported by Magon in India [15]. However, amebic infection is not often suspected in very young children even in the endemic areas.

The definitive diagnosis of intestinal amoebiasis is made by the demonstration of haematophagous trophozoites of *E. histolytica* [16]. It is difficult to distinguish *E. histolytica* from *Entamoeba dispar* or *Entamoeba moshkovaskii* infection. Therefore, the stool should be examined for specific antigen or DNA and also serum test for antiamebic antibodies. This also should have been done for the mother. In this case, stool examination revealed cyst and trophozoites. There was associated diarrhea in the clinical presentation. The results of laboratory exams were positive. Hence, the present case represents a rare form of amebic infection (infantile amoebiasis).

## 4. Conclusion

In conclusion, to our knowledge, this is the first reported case of infantile amoebiasis in our area. It draws attention to the possibility of encountering amoebiasis in infant. Such cases should be monitored more closely. An early precise diagnosis is of prime importance because appropriate treatment in addition to supportive care can be life-saving for such patients.

## Figures and Tables

**Table 1:** The patient's laboratory test results.

Indicator	The patient's values	Normal
White blood count (×1000/*μ*L)	11.2	4.0–10.0
Red blood count (×10^6^/*μ*L)	4.5	4.6–6.0
Eosinophil (%)	7.0	1–10
Hemoglobin concentration (g/dL)	11.6	11.5–18
Na (mEq/L)	130.0	135–145
K (mEq/L)	4.8	3.9–5.5
Ca (mg/dL)	10.4	10.0–12.0
P (mg/dL)	5.4	4.0–8.0
Serum total protein (g/dL)	4.6	4.5–7.5
CRP	Positive	Negative
